# Validation of Reference Genes in *Solenopsis invicta* in Different Developmental Stages, Castes and Tissues

**DOI:** 10.1371/journal.pone.0057718

**Published:** 2013-02-28

**Authors:** Daifeng Cheng, Zhiling Zhang, Xiaofang He, Guangwen Liang

**Affiliations:** Department of Entomology, South China Agricultural University, Guangzhou, Guangdong, People’s Republic China; Swedish University of Agricultural Sciences, Sweden

## Abstract

To accurately assess gene expression levels, it is essential to normalize real-time quantitative PCR (RT-qPCR) data with suitable internal reference genes. For the red imported fire ant, *Solenopsis invicta,* reliable reference genes to assess the transcript expression levels of the target genes have not been previously investigated. In this study, we examined the expression levels of five candidate reference genes (*rpl18*, *ef1-beta*, *act*, *GAPDH*, and *tbp*) in different developmental stages, castes and tissues of *S. invicta*. To evaluate the suitability of these genes as endogenous controls, three software-based approaches (geNorm, BestKeeper and NormFinder) and one web-based comprehensive tool (RefFinder) were used to analyze and rank the tested genes. Furthermore, the optimal number of reference gene(s) was determined by the pairwise variation value. Our data showed that two of the five candidate genes, *rpl18* and *ef1-beta,* were the most suitable reference genes because they have the most stable expression among different developmental stages, castes and tissues in *S. invicta*. Although widely used as reference gene in other species, in *S. invicta* the act gene has high variation in expression and was consequently excluded as a reliable reference gene. The two validated reference genes, *rpl18* and *ef1-beta,* can be widely used for quantification of target gene expression with RT-qPCR technology in *S. invicta*.

## Introduction

The use of reference gene(s) as internal control for the measurement of target gene expression variation is the currently preferred method for normalizing real-time quantitative PCR (RT-qPCR) data because reference genes can capture all non-biological variations [1]. Although no gene exhibits constant expression under all experimental conditions, studies validating reference genes have been driven by several algorithms and freely available softwares (e.g., geNorm [2], Bestkeeper [3], NorFinder [4] and RefFinder [http://www.leonxie.com/referencegene.php?type=reference]) which can analyze the stability of gene expression.

To date, several genes, such as ribosomal protein L18 (*rpl18*) [5]–[7], translation elongation factor 1 (*ef1-beta*) [8], [9], actin (*act*) [8], [9], glyceraldehyde-3-phosphate dehydrogenase *(GAPDH)* [8], [9] and TATA box binding protein (*tbp*) [10] have been used widely as candidate reference genes for gene expression studies in Insecta and other groups of organisms [11], [12]. However, no universal reference gene that is stable under all experimental circumstances has been described yet [13]. The expression of reference genes can differ widely in their stability over developmental stages [13]–[16], in different tissues [17]–[21] and under different environmental conditions [14], [22], [23]. Therefore, a rational optimization approach to select and standardize reference genes is now an important requirement in RT-qPCR-based transcriptome studies [24].

The red imported fire ant, *Solenopsis invicta* Buren, is a notorious invasive species [25], [26] which has caused biodiversity declines, disrupted ecosystem functions and incurred severe socio-economic losses worldwide [27]. The increasing numbers of hypersensitivity reactions to *S. invicta* sting sequelae represent a public health concern [28]. Moreover, recent genome and transcriptome studies have thoroughly investigated the evolutionary history of this invasive pest and made available genetic tools (i.e. microarray [29] and genome [30]), while setting *S. invicta* as a model organism for the evolution of castes and polyphenism [31]–[34]. RT-qPCR can therefore represent a very useful mean to further investigate the expression pattern of selected target genes.

There is no validated reference gene(s) for *S. invicta*. In previous RT-qPCR studies on *S. invicta*, reference genes (e.g., *18 s* [35] and *ß*-actin [36]) were selected based on consensus or results from other species. Though four housekeeping genes (*rpl49, ef1-beta, act* and *GAPDH*) were suitable genes for normalization in honey bee, *Apis mellifera* [8], [9], which is also eusocial insect having close relationship to *S. invicta*, stability of their expressions were examined only in larval and pupal stages [8]. The adult stage should be an important developmental stage to be probed since RNA transcript profiles can change during eclosion process from pupa to adult stage [32]. In addition, as reported in previous studies, gene expression profiles in *S. invicta* are influenced by developmental stages [32], castes (e.g., alate and dealate queen; the queen- and worker-specific expression) [30], [37] and tissue types [38]. Thus, a systematic validation of reference gene is required to ensure proper normalization in *S. invicta*.

Our aim of this work is to identify suitable reference gene(s) and minimize the risk of coregulation artifacts in *S. invicta*. Expressions of five candidate reference genes (*rpl18*, *ef1-beta*, *act, GAPDH* and *tbp*) were examined in three developmental stages (larval, pupae and adult), three castes (worker, alate female and alate male) and five tissues from the following parts of the insect body: antenna, head without antenna, thorax, leg and abdomen. Furthermore, the consistency of the best-scoring reference gene was tested by four statistical approaches (geNorm, BestKeeper, NormFinder and RefFinder).

## Materials and Methods

### Biological Samples

Fifteen colonies of *S. invicta* (broods, workers, alates and queens) were collected with soil from the campus of South China Agriculture University, Guangzhou, China. Colonies were transferred into a 30 L plastic box coated with talcum powder to prevent escape. Ants were fed with 10% sugar solution and mealworms at 25±2°C and 50±5% relative humidity in the laboratory. Among our colonies there was sex-bias in the production of alates [39], with only five of fifteen colonies having both alate females and males. Thus, gene expression profiles were analyzed in three developmental stages (larva, worker pupa and worker) and three castes (worker, alate female and alate male) from these five colonies. Each sample used for developmental stages and castes analyses consisted of five individuals that were picked directly from the five colonies by soft forceps and flash-frozen in a −40°C freezer. Since the tissues from individuals are too small (e.g. antennae) to use for RNA quantification and RT-qPCR, tissue samples (the antenna, head (without antenna), thorax, leg and abdomen) were separately dissected from more than 200 workers of each colony and immediately pooled into single tubes containing RNA storage solution (Qiagen) then stored at −80°C.

### Total RNA Extraction

Total RNA was extracted using TRIzol® (Invitrogen), and dissolved in RNase-free water and stored at −80°C. The RNA integrity was determined by denaturing agarose gel (1.2%) electrophoresis in 1x TAE buffer (0.04 M Tris-acetate, 0.00l M EDTA [pH 8.0]) and stained by GoldviewTM. The integrity of the RNA was confirmed by agarose gel electrophoresis, showing intense ribosomal RNA bands with absence of smears. The RNA concentration of each RNA sample was measured in triplicate using a Nanodrop2000 spectrophotometer (Thermo, USA). The purity of the total extracted RNA was measured by the 260/280 nm ratio, with expected values between 1.8 and 2.0. The concentration of total RNA was standardized to 2 µg/mL.

### Primer Design

The primers were designed using NCBI Primer-BLAST (http://www.ncbi.nlm.nih.gov/tools/primer-blast/). To achieve high specificity of the target genes, we ensured that the primers had at least 2 mismatches to non-target genes in the *S. invicta* genome. The gene characteristics and primer sequences were summarized in Table 1. Sequences of five candidate genes from *S. invicta* were acquired by EST search in the Genbank. To identify the putative functional genes, BLAST was applied to search for five candidate genes against the GenBank Nucleotide collection database of *A. mellifera*. All the putative functional genes from *S. invicta* genome with homologyapp:addword:homology above 69% were deemed to have the same function as the corresponding gene from *A. mellifera*.

**Table 1:** Sequence information for five selected candidate reference genes.

Gene symbol	Primer sequence (5′→3′)	Accession number	Function	Amplification efficiency
*rpl18*	Forward: GCATGATCGGAAAGTGCG	EH413666	Structural constituent of ribosome	98.7%
	Reverse: TTCAGCCACTTGACTGCG			
*ef1-beta*	Forward: TGAAGACCGATAAGGGCA	EH413796	Elongation during polypeptide synthesis at the ribosome	95.6%
	Reverse: TCGTCCGAACCAAAGAGA			
*act*	Forward: CTGTCCCGTGCACGAGCGTT	EH413679	Involved in cell motility, structure and integrity	93.5%
	Reverse: GGGAAAGACGGCGCGAGGAG			
*tbp*	Forward: CGACTTTGTATCGTTTCTCG	EH413121	Transcription initiation from RNA polymerase II promoter	101%
	Reverse:TTATACGGACGCACTTCATC			
*GAPDH*	Forward: AAGCTGTGGCGTGATGGCCG	EH413647	Carbohydrate metabolism	104%
	Reverse: AGGAGGCAGGCTTGGCGAGT			

### cDNA Synthesis and Sequencing

After the RNA samples were treated with DNAse, cDNAs were synthesized from 1 µg of total RNA in triplicate by reverse transcription catalyzed by PrimerScript® reverse transcriptase (Invitrogen) and an oligo (dT) 12−18 primer. PCR amplification was performed using the following program: 2 min at 94°C followed by 30 cycles of 30 s at 94°C, 30 s at 60°C, 30 s at 72°C and a final extension step for 10 min at 72°C. Following amplification, the PCR products were evaluated by running 2 µl of the product on 1% agarose gels. Amplified products with correct (predicted) size were incised from agarose gels and purified. Gene identity was then confirmed by sequencing using an ABI 3730×l sequencer (BGI Life Tech Co. Ltd., Shenzhen, China). Sequences with 99% or more similarity to the target genes were used as templates for the real-time PCR assays. Meanwhile, the synthesized cDNAs were stored at −20°C for further RT-qPCR.

### Standard Curve Construction and Real-time qPCR

To ensure the reliability of the amplification results, the RT-qPCR amplification efficiencies were determined by the standard curve and melting curve analysis [35]. Standard curves were constructed by using 10-fold serially diluted cDNA as a template prior to candidate gene expression analysis. Every template used in RT-qPCR amplification was analyzed by three independent runs. The threshold cycle (*Ct*) values were plotted against log transformed concentrations of serial dilutions to obtain the slope. *Ct* values for each gene were determined by the arithmetic mean of the three values. Only genes with amplification efficiency between 90% and 110% were used for further statistical analysis. Gene expression analyses were conducted on an Agilent Technologies Stratagene M×3005P by real time quantitative PCR using the SYBR®GREEN method. Each sample was measured in three independent replicates with a total reaction volume of 25 µL containing 0.5 µL of each primer (diluted to 10 µM), 12.5 µL SYBR® Premix Ex Taq™, 9.5 µL ddH_2_O and 2 µL template. Cycling conditions were as follows: 95°C for 10 min and 40 cycles of 95°C for 30 s, 60°C for 45 s and 72°C for 1 min, followed by a final melting curve analysis. For every gene analyzed, template controls were excluded to detect possible contamination. The specificity of the PCR products was confirmed by melting curve analysis for all samples.

### Data Analysis and Statistics

Three software programs, geNorm (version 3.4) [2], BestKeeper (version 1) [3], and NormFinder (version 0.953) [4] were used to evaluate the stability of the candidate reference genes. Both geNorm and Bestkeeper use a pairwise comparison approach. The geNorm program calculates a gene stability measure (*M*) value and a pairwise variation (*V*) value to determine the most stably expressed gene and the optimal number of reference genes, respectively. The gene with the lowest *M* value should be the most stably expressed gene. To estimate the optimal number of reference gene(s), we used pairwise variation *V*_n/n+1_, which was calculated between the two sequential normalization factors (NF_n_ and NF_n+1_) by geNorm to determine the minimum number of reference genes. The n indicated the number of the most stable reference genes. Vandesompele et al. (2002) proposed 0.15 as a cut-off value for the *V* value below which the inclusion of an additional control gene is not required.

Likewise, the Bestkeeper index, which is based on the geometric mean of the Ct values of the candidate reference genes with a standard deviation (SD), was used to rank reference genes. Lower index scores denote greater transcriptional stability and thus better suitability as a reference gene.

NormFinder uses a model-based approach to estimate expression variation in selection of suitable reference genes [4]. Genes with the lowest values are the most stable genes.

RefFinder was used to evaluate and screen optimal reference genes by integrating the results of the previous three analyses. Multiple comparisons of *Ct* values were performed by analysis of variance (One-Way ANOVA) followed by followed by Tukey Kramer HSD for at the 95% confidence level in SPSS Statistics version 19 (IBM Corp., Somers, NY). Finally, we obtained a ranking of the candidate genes determined by RefFinder to choose the most stable genes.

## Results

### qPCR Assay Validations

All PCR assays produced a single amplicon, as shown by the absence of nonspecific bands in electrophoresis analysis and the presence of a single sharp peak in melting curve analysis. All PCRs displayed efficiency between 93.5% and 104%, as shown in Table 1. PCR efficiency values between 90–110% are considered acceptable (Agilent Technologies Stratagene).

### Transcript Profiles of Reference Genes

**Figure 1:**
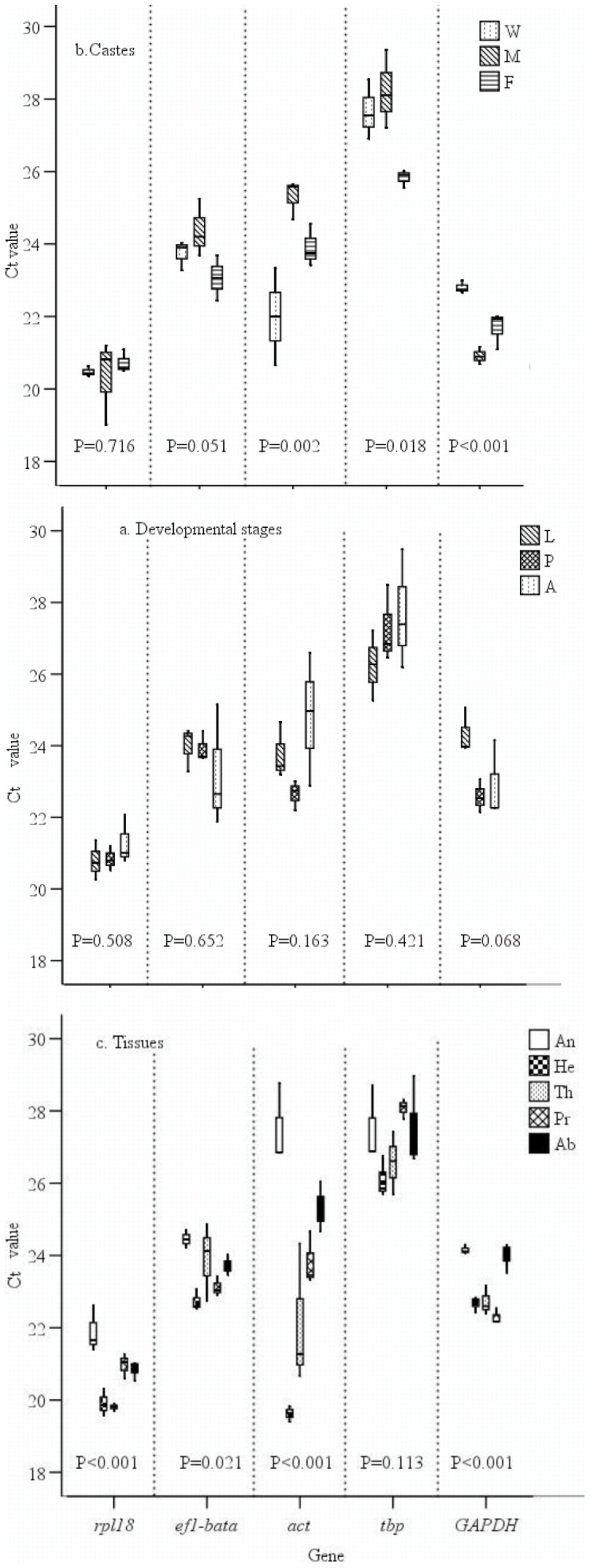
Threshold cycles (Ct) comparison of five reference genes. a: Developmental stages; b: Castes; c: Tissues. *P* values are given for each of the five putative genes (One-Way ANOVA). Developmental stages: Larvae (L), Pupae (P), Adult (A); Castes: Worker (W), Alate Male (M), Alate Female (F); Tissues: Antenna (An), Head without antennae (He), Thorax (Th), Leg (Pr) and Abdomen (Ab).

The expression profiles of the five candidate reference genes were assessed in different developmental stages, castes and tissues (Figure 1a, 1b, 1c). The distributions of the *Ct* values for each gene in samples from different developmental stages were not significantly different, with probability values (*P)* greater than 0.068 (One-Way ANOVA, Figure 1a). Two of five candidate genes, *rpl18* and *ef1-beta* had no significantly different *Ct* values between castes, while other three candidate genes, *act*, *tbp* and *GAPDH* had significantly different *Ct* values between castes with *P*≤0.018 (One-Way ANOVA, Figure 1b). The *Ct* values for *rpl18*, *ef1-beta*, *act* and *GAPDH* were significantly different among different tissues (One-Way ANOVA, *P*≤0.021), whereas no significant differences were detected in *tbp* expression in different tissues (Figure 1c).

### The Optimal Number of Reference Genes

**Figure 2:**
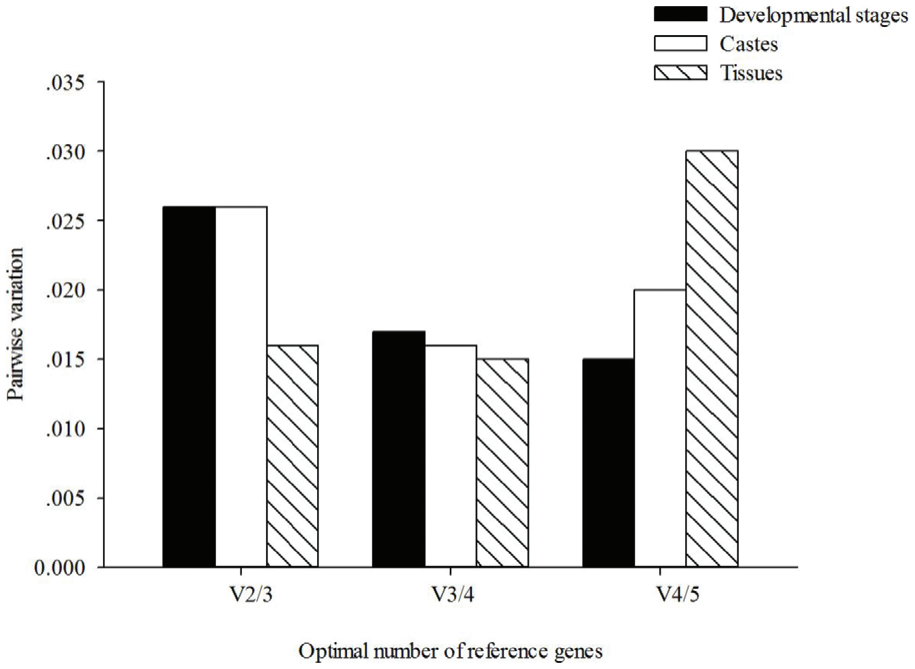
The optimal number of reference genes for normalization by geNorm analysis.

Pairwise variation values were determined individually for the developmental stages, castes and tissues datasets. Results showed that pairwise variation values for *V*_2/3_ were below the cut-off value of 0.15 in all scenarios (Figure 2). Thus, no third control gene was required when RT-qPCR was carried out in any of the three treatments, meaning that the use of two most stable candidate genes (i.e., *rpl18* and *ef1-beta*) as reference genes would be sufficient for a stable and valid reference in RT-qPCR analysis.

### Identification of the Optimal Reference Genes

Analyses using four programs ranked the tested genes according to the determined control gene stability measure from the most stable (lowest value) to the least stable (highest value), as shown in Table 2. All stable values acquired by geNorm, BestKeeper, NormFinder were summarized in Table S1. For genes other than *act*, BestKeeper revealed an overall stability in gene expression with SD values less than 1, while the values for *act* were greater than 1 in all three datasets. These results suggest that the expression of *act* is not stable and should be excluded.

**Table 2:** Rank of reference genes based on their expression stability in different developmental stages, castes and tissues of *S. invicta* according to geNorm, BestKeeper, NormFinder and RefFinder analysis.

	geNorm			BestKeeper			NormFinder			RefFinder		
	Developmental stages	Castes	Tissues	Developmental stages	Castes	Tissues	Developmental stages	Castes	Tissues	Developmental stages	Castes	Tissues
Most stable gene	*tbp*	*ef1-beta*	*rpl18*	*rpl18*	*rpl18*	*ef1-beta*	*rpl18*	*ef1-beta*	*rpl18*	*ef1-beta*	*rpl18*	*rpl18*
↑	*ef1-beat*	*rpl18*	*ef1-beta*	*ef1-beta*	*ef1-beta*	*rpl18*	*ef1-beta*	*rpl18*	*GAPDH*	*rpl18*	*ef1-beta*	*ef1-beta*
	*rpl18*	*tbp*	*GAPDH*	*GAPDH*	*GAPDH*	*GAPDH*	*GAPDH*	*tbp*	*ef1-beta*	*GAPDH*	*GAPDH*	*GAPDH*
↓	*GAPDH*	*GAPDH*	*tbp*	*tbp*	*tbp*	*tbp*	*tbp*	*GAPGH*	*tbp*	*tbp*	*tbp*	*tbp*
Least stable gene	*act*	*act*	*act*	*act*	*act*	*act*	*act*	*act*	*act*	*act*	*act*	*act*

Across different developmental stages, the order of gene expression stability (from the most stable to the least stable) was calculated as *tbp*, *ef1*-*beta*/*rpl18* (equivalent) *GAPDH* and *act* by geNorm and as *rpl18, ef1-beta, GAPDH, tbp*, and *act* by Bestkeeper and Normfinder (Table 2). All three programs gave the same results for the caste comparisons, indicating that *ef1-beta* and *rpl18* were the most stable expressed genes. Across different tissues tested, geNorm and Bestkeeper identified *ef1*-*beta* and *rpl18* as the most stable expressed genes, while Normfinder selected *rpl18* and *GAPDH*. Moreover, integrating results of previous three analyses, RefFinder confirmed that *rpl18* and *ef1-beta* were the most stable expressed genes among all these three datasets (developmental stages, castes and tissues). Considering that the *V*2/3 values were below 1.5, two genes (*rpl18* and *ef1-beta*) were recommended for use as reference genes (Figure 2).

## Discussion

To our knowledge, this is the first systematic study to validate a set of candidate reference genes for RT-qPCR in *S. invicta*. Our results suggested that *rpl18* and *ef1-beta* were the best reference genes according to the average expression stability (*M*) or stability values acquired by geNorm, BestKeeper, Normfinder and RefFinder. When the outcomes of four algorithms were compared, only few differences were observed except for the samples from developmental stages and tissues. Considering the developmental stages, the most stable genes were *tbp/ef1-beta* (geNormal), *ef1-beta/rpl18* (Bestkeeper, NormFider and RefFinder), respectively. Among different castes, *rpl18* and *ef1-beta* had constant expression in all data sets produced by the four algorithms. For different tissues, *rpl18* and *ef1-beta* were identified as the two most stable genes by geNorm, Bestkeeper and RefFinder, while the two most stable genes by NormFinder analysis were *rpl18* and *GAPDH*. Based on the rankings from RefFinder, which integrates outcomes of three major statistic algorithms (geNorm, Normfinder and Bestkeeper) and assigns an appropriate weight to an individual gene and calculates the geometric mean of their weight, *rpl18* and *ef1-beta* genes had a good performance under specific conditions. Moreover, *ef1* (including *ef1-alpha* and *ef1-beta*) had also been proved to be the best candidate reference gene for normalization in emerald ash borer [40], triatomines [41], bed bug [6] and honey bee [8].

When comparing our results with reference genes of honey bee *A. mellifera*, we observed that four of the genes analyzed in *S. invicta* are putative homologues of the best reference genes in *A. mellifera* (*rpl49, ef1-beta*, *act* and *GAPDH*) [8]. However, expression of genes *act* and *GAPDH* were too unstable to be used for normalization in *S. invicta.* Despite systematically well scored in desert locust [42], planthoppers [43] and honey bee [8], *act* was excluded as a reference gene in *S. invicta* because it had the largest *M* value (geNorm) and an SD value (Bestkeeper) greater than 1. The *act* gene exhibited high variation in expression among different castes and tissues with *P*  = 0.002 and *P*<0.001, respectively. These results are in accordance with several studies demonstrating *act* gene as an unsuitable internal control for RT-PCR in other cell types or species [40], [44]. Recent studies have shown that *β-act* (one of the three main groups of *act* isoform, α, β and γ) expression can change in response to variety of conditions [45]. Thus, these traditionally used reference genes are not always stably expressed in broad species or experimental treatments [46], [47], which also emphasizes the need to evaluate reference gene in *S. invicta*.

The expression profiles of candidate genes were more variable among castes and tissues than between developmental stages in *S. invicta*. Based on our results, the five candidate genes exhibited no significant differences across developmental stages with *P*>0.05, while three (*act*, *GAPDH*, and *tbp*) and four (*rpl18*, *ef1-beta*, *act*, and *GAPDH*) of five candidate genes exhibited significant differences in expression among different castes (*P*<0.05) and tissues (*P*<0.05). Transcriptome microarray hybridizations experiments indicated on the other hand that gene expression profiles were influenced more by developmental stages than by castes [31], [32], Thus the reference genes identified in the present study may be particularly useful for intra-caste analyses of gene expression.

In conclusion, we tested the suitability of five genes with four commonly used programs and confirmed that *rpl18* and *ef1-beta* are the most suitable reference genes to investigate the gene expression profiles in developmental stages, castes and tissues for *S. invicta*. Furthermore, these findings may also be useful for Northern blot and reverse transcription PCR techniques that require reference gene for normalization. The validation of reference genes in our study provides new information that will be useful for the accurate elucidation of the expression profiles of target genes in *S. invicta*.

## Supporting Information

Table S1 **Ranking of reference genes by stable values of** ***S. invicta.*** Genes were ranked by the stable values obtained with three softwares (geNorm, BestKeeper, Normfinder) based on the gene expression stability in different developmental stages, castes and tissues. M value: stability measure value. The gene with the lowest M value denotes the most stably expressed gene. SD mentioned standard deviation of threshold cycle (*Ct*) value. Any studied gene with the SD higher than 1 can be considered inconsistent and not suit to be reference gene. Stability value: Lower scores denote greater transcript stability and suitability as a reference gene.
(DOC)
