# Supplementary material for: Validation of Reference Genes in Solenopsis invicta in Different Developmental Stages, Castes and Tissues
Source: PLoS One. 2013 Feb 28;8(2):e57718. doi: 10.1371/journal.pone.0057718 (PMC3585193; doi:10.1371/journal.pone.0057718)
Supplement: Table S1 — Ranking of reference genes by stable values of S. invicta. Genes were ranked by the stable values obtained with three softwares (geNorm, BestKeeper, Normfinder) based on the gene expression stability in different developmental stages, castes and tissues. M value: stability measure value. The gene with the lowest M value denotes the most stably expressed gene. SD mentioned standard deviation of threshold cycle (Ct) value. Any studied gene with the SD higher than 1 can be considered inconsistent and not suit to be reference gene. Stability value: Lower scores denote greater transcript stability and suitability as a reference gene. (DOC) [file pone.0057718.s001.doc]

Table S1 **Ranking of reference genes by stable** values of *S. invicta*

| Approaches | Developmental stages | | Castes | | Tissues | |
| --- | --- | --- | --- | --- | --- | --- |
|  | Genes | Values | Genes | Values | Genes | Values |
| geNorm  (M value) | *tbp*  *ef1-beta*  *rpl18*  *GAPDH*  *act* | 0.080  0.083  0.083  0.092  0.109 | *ef1-beta rpl18*  *tbp*  *GAPDH*  *act* | 0.077  0.079  0.081  0.081  0.088 | *rpl18*  *ef1-beta*  *GAPDH*  *tbp*  *act* | 0.076  0.079  0.079  0.091  0.154 |
| BestKeeper  (SD [±*Ct*]) | *rpl18*  *ef1-beta*  *GAPDH*  *tbp*  *act* | 0.39  0.756  0.902  0.959  1.112 | *rpl18*  *ef1-beta*  *GAPDH*  *tbp*  *act* | 0.361  0.487  0.700  0.971  1.15 | *ef1-beta*  *rpl18*  *GAPDH*  *tbp*  *act* | 0.655  0.694  0.727  0.857  2.41 |
| Normfinder (Stability value) | *rpl18 ef1-beta*  *GAPDH*  *tbp*  *act* | 0.813  0.827  (0.972  1.090  1.169 | *ef1-beta rpl18*  *tbp*  *GAPGH*  *act* | 0.420  0.818  0.963  1.182  1.548 | *rpl18*  *GAPDH*  *ef1-beta*  *tbp*  *act* | 0.378  0.558  0.616  1.119  2.398 |

Genes were ranked by the stable values obtained with three softwares (geNorm, BestKeeper, Normfinder) based on the gene expression stability in different developmental stages, castes and tissues. M value: stability measure value. The gene with the lowest M value denotes the most stably expressed gene. SD mentioned standard deviation of threshold cycle (*Ct*) value. Any studied gene with the SD higher than 1 can be considered inconsistent and not suit to be reference gene. Stability value: Lower scores denote greater transcript stability and suitability as a reference gene.
